# CpG Distribution and Methylation Pattern in Porcine Parvovirus

**DOI:** 10.1371/journal.pone.0085986

**Published:** 2013-12-31

**Authors:** Renáta Tóth, István Mészáros, Rajmund Stefancsik, Dániel Bartha, Ádám Bálint, Zoltán Zádori

**Affiliations:** 1 Institute for Veterinary Medical Research, Centre for Agricultural Research, Hungarian Academy of Sciences, Budapest, Hungary; 2 Division of Genetics, FlyBase, University of Cambridge, Cambridge, United Kingdom; 3 National Food Chain Safety Office, Veterinary Diagnostic Directorate, Budapest, Hungary; University of Kansas Medical Center, United States of America

## Abstract

Based on GC content and the observed/expected CpG ratio (oCpGr), we found three major groups among the members of subfamily *Parvovirinae*: Group I parvoviruses with low GC content and low oCpGr values, Group II with low GC content and high oCpGr values and Group III with high GC content and high oCpGr values. Porcine parvovirus belongs to Group I and it features an ascendant CpG distribution by position in its coding regions similarly to the majority of the parvoviruses. The entire PPV genome remains hypomethylated during the viral lifecycle independently from the tissue of origin. *In vitro* CpG methylation of the genome has a modest inhibitory effect on PPV replication. The *in vitro* hypermethylation disappears from the replicating PPV genome suggesting that beside the maintenance DNMT1 the *de novo* DNMT3a and DNMT3b DNA methyltransferases can’t methylate replicating PPV DNA effectively either, despite that the PPV infection does not seem to influence the expression, translation or localization of the DNA methylases. SNP analysis revealed high mutability of the CpG sites in the PPV genome, while introduction of 29 extra CpG sites into the genome has no significant biological effects on PPV replication *in vitro*. These experiments raise the possibility that beyond natural selection mutational pressure may also significantly contribute to the low level of the CpG sites in the PPV genome.

## Introduction

DNA methylation is the prime form of epigenetic modifications of the eukaryotic genome. In vertebrate cells almost exclusively the 5^th^ carbon atom of cytosine is methylated within CpG dinucleotides. Methylation has a significant impact on chromatin structure modulation, genomic imprinting and X chromosome inactivation. It can inhibit transcription by preventing the binding of transcription factors or by recruiting methyl-binding proteins and histone deacetylases leading to the formation of condensed chromatin structure [1]. 

In mammals approximately 60-90% of CpGs comprise methylated cytosine bases [2]. The 5’ end of the housekeeping genes are often associated with a GC-rich stretch of DNA containing high amounts of CpG dinucleotides, so called CpG islands, which are free of methylation [3]. DNA methyltransferases (DNMTs) are responsible for the conversion of cytosines to 5-methylcytosines. The DNMTs are divided into two groups: maintenance (DNMT1) and *de novo* (DNMT3a and DNMT3b) methyltransferases. The mechanism of site specific CpG methylation and regulation of DNMTs to develop specific patterns are presently not well understood [4].

CpGs are observed only at one-fourth to one-third of their expected frequency [5,6] in most vertebrate genomes. Several explanations have been proposed to account for this discrepancy, deamination and timine conversion of methylated cytosines, avoidance of higher stacking energy of CpG dinucleotides during replication and prevention of autoimmune reactions among others.

Unmethylated CpGs (UCpGs) as signature of invading bacterial and viral organisms are immunostimulants even on short oligonucleotides in mammals. Immune response is triggered by the UCpGs binding to TLR9, a member of the Toll-like receptor family on the surface of dendritic cells [7]. Therefore CpG methylation is not only important in the regulation of the hosts’ life processes, but it also plays a key role in the detection of microbial and viral pathogens and inactivation of integrated foreign DNA [8,9], consequently it has major influence on the lifecycles of DNA- and retroviruses as well. The role of methylation in viral regulation is less understood than in mammals. Integrated adenoviruses and papovaviruses are generally hypermethylated, while the actively replicating viral DNA is hypomethylated with methylated sites in specific regions of the viral genome [9]. EBV is highly methylated during latency, and becomes demethylated during active replication. It uses methylation-induced gene silencing to evade host immunity [10]. In contrast, ranid herpesviruses are heavily methylated during replication and probably code their own DNA cytosine-5 methyltransferases [11]. CpG dinucleotides are underrepresented in most of the small DNA viruses. This pattern is thought to be established by evolutionary pressure to avoid CpG-mediated immune responses and to decrease the direct interference of methylation on the transcription of viral RNAs and viral replication [9].

Parvoviruses are small single stranded DNA viruses with an approximately 4-6 Kb linear genome. Despite their small genome and their limited coding capacity parvoviruses are surprisingly successful to invade a wild variety of host organisms from insects to mammals and constitute a large, diverse virus family [12]. Their diversity manifests not only in the large number of parvoviral species, but also in the complexity of their lifecycle. Beside lytic infection some parvoviruses are able to infect their respective host persistently [13,14]. Adeno-associated viruses are able to insert their genome into the host genome to establish latent infection and subsequently are capable to parasitize the transcription machinery of other viruses and reactivate their own replication mechanism during helper virus infection [15]. 

Members of the *Parvovirinae* subfamily are among the most dangerous and economically most harmful viruses of domesticated and farm animals (e.g., canine parvovirus (CPV) [16], goose parvovirus (GPV) [17] porcine parvovirus (PPV) [18] and are also able to cause diseases in humans (B19) [19] and human bocavirus [20,21]. PPV is responsible for syndrome of reproductive failure in swine, included infertility, early embryonic death, mummified fetuses and stillbirths [22]. It frequently causes persistent infection combined with chronic shedding [23]. 

There are some well-established connections between persistent viral infection and CpG methylation in other virus families [24,25] but not much is known about the role of epigenetic modifications in parvoviruses. In this paper our aim is to expand our knowledge about the effect of CpG methylation in the life cycle of parvoviruses focusing on PPV and to reveal whether methylation has any direct influence on the evolution of the CpG poor PPV genome. 

Our *in silico* analysis of the CpG pattern of parvoviruses revealed that parvoviral genomes are more heterogenic in their CpG contents than it was previously recognized and a group of parvoviruses exists in which CpGs are not depleted despite that their genomes are AT rich. PPV DNA was found hypomethylated independently from its tissue origin. In vitro methylation of PPV DNA or the introduction of additional CpG sites into the PPV genome had no significant effect on PPV replication in vitro. These data indicate that CpG methylation has no regulating role in PPV life cycle and together with the recently published findings that parvoviruses do not induce TLR9 activated immune response [26] suggest that CpG depletion in the genome of PPV and other parvoviruses is most probably the consequence of other evolutionary forces than CpG methylation.

## Materials and Methods

### Computing CpG distribution in coding positions and SNP analysis

Parvovirus sequences have been collected from the NCBI nucleotide databank (Table S1a). The contiguous protein coding sequences for *Drosophila melanogaster* were downloaded in FASTA format from the FTP site of FlyBase (release r5.24) [27]. The core set of human coding sequences are from The Consensus CDS (CCDS) project [28]. The protein coding regions of the human mRNAs were downloaded in FASTA format from the CCDS database [29]. Dinucleotide frequencies as a function of coding sequence position were calculated using custom Bash scripts (available upon request). Single nucleotide polymorphism (SNP) was calculated from 68 PPV sequences containing the full coding regions or the complete NS or VP genes (Table S1b) with a custom made program. The algorithm counts the polymorphism sites (distinguishing the transition events at C, G, CpG and GC nucleotides) in a multiple alignment where a consensus base is only considered with 75% confidence or above (C++ source code available upon request).

### Viral propagation

PT (porcine testis) [30] and Cos 7 (African green monkey kidney) [31] cell lines were used for the propagation of the NADL-2 strain of PPV and its derivatives. Cells were grown in Dulbecco’s modified Eagle’s medium with high glucose (4,5 g/l) and L-glutamine (PAA, Cölbe, Germany) supplemented with 1% PenStrep (100X; penicillin: 10 000 U/ml, streptomycin: 10 mg/ml; PAA), 1% sodium-pyruvate solution (100 mM; Lonza, Basel, Switzerland) and 10% Fetal Bovine Serum Gold (PAA).

### Viral DNA extraction and determination of the methylation pattern

The packaged form of the viral DNA was extracted from 1 ml tissue supernatant by the High Pure Viral Nucleic Acid Kit (Roche, Basel, Switzerland) according to the manufacturer’s recommendations. The replicative PPV DNA was purified by using modified Hirt extraction [32] as it is described by Molitor et al. [33]. The methylation status of the viral DNAs was determined by bisulfite PCR, cloning and sequencing. For the bisulfite conversion the EpiTect Bisulfite Kit (Qiagen, Venlo, Netherlands) was used according to the manufacturer’s instructions. The modified CpG containing DNA fragments of the positive and negative strands were amplified by PCR primers (Table 1) which were designed using MethPrimer program [34]. PCR reactions were executed by DreamTaq DNA Polymerase (Thermo Fisher Scientific, Waltham, MA, USA). The DNA amplifications were carried out by initial denaturation for 5 min at 95 °C, followed by 30 cycles at 95 °C for 20 s, 52 °C for 20 s, and 72 °C for 20 s. The amplified DNA fragments were cloned into pJET1.2 blunt cloning vector (Thermo Fisher Scientific) and sequenced by using BigDye Terminator v3.1 Cycle Sequencing Kit (Applied Biosystems, Foster City, CA, USA) following the instructions of the manufacturers.

**Table 1:** PCR primers for the amplification of bisulfite treated PPV DNA.

**Primer name**	**Sequence**	**Product size (bp)**	**CpGs in product**		**Primer name**	**Sequence**	**Product size (bp)**	**CpGs in product**
MET1F	5'-ATTTTTAAATTGATTAATTGTTTTTG-3'	197	12		MREV1F	5'-TTTATATGATTTTATGTTTTTAGGGTGG-3'	157	7
MET1R	5'-ATAATATTAAACTCCACCTCTTTTT-3'				MREV1R	5'-AATAAAAATTACTATATATTCCTTTAAATT-3'		
MET3F	5'-TTTTTTTTGTTTTAGATTGTATTT-3'	258	4		MREV3F	5'-TTTTTAGGTGTTGTTGGTGTGTAT-3'	334	5
MET3R	5'-ACTCCTCTTTATAAATTTATCATTTCC-3'				MREV3R	5'-AAAAAATACATATACTAAATTCAAAATCAA-3'		
MET4F	5'-TTGTTTGGAATAATTATAATAAAGATATAA-3'	154	3		MREV4F	5'-TTATTTTTGATTTTGAATTTAGTATATGTA-3'	262	5
MET4R	5'-AAAAAATCAAAAATCAATACTTACC-3'				MREV4R	5'-AAACAACACAACCCTATTAATACAAAC-3'		
MET5F	5'-AATAATGTAATGTAAAGTATTTTTAATATT-3'	341	3		MREV5F	5'-ATATGTTTTGGAGTAGGTTTTTTTT-3'	232	5
MET5R	5'-TTTTAACTTCCTAAACTATAATAACC-3'				MREV5R	5'-ATCCATACTTCTACTTCTCAACAACTAATA-3'		
MET6F	5'-ATAAATTTGAAATTGTGGAAATAA-3'	262	2		MREV6F	5'-TGTTTTGAAGAAGTAATGTTTAATTTTATT-3'	190	7
MET6R	5'-AAATCATATACTATTTTTATTCTTACTAAA-3'				MREV6R	5'-ACTAAACCAAAAAAAACCAACTAATC-3'		
MET7F	5'-AATTTTTGTAATAATGATTATAAATGAAGA-3'	143	1		MREV7F	5'-TATTTTTTGTTTTTTTTGTAGGAGG-3'	224	7
MET7R	5'-AAAATCCAAAATCACCTAACAATTTT-3'				MREV7R	5'-CCTAAATTTAACTTTAACCTTAAAACC-3'		
MET8F	5'-GATTTTTAGATTTTTATATTAGTGAAAATT-3'	297	10		MREV8F	5'-GGTTTTTTTGTATTGGAGTTGTTG-3'	113	3
MET8R	5'-ACCTCTTACTCTTTTTACAAAAAAC-3'				MREV8R	5'-AAAACCAAAAATACAAACCCCA-3'		
MET9F	5'-TAAGGAGAATTAATTAATTTATTAGA-3'	359	10		MREV9F	5'-TTTTTTTTAAAAGTTTAAAATTATTTGGTA-3'	173	1
MET9R	5'-ATATTAAATATCCCTTTAACTTTTT-3'				MREV9R	5'-AAAAAACAAACAAATTAAACCAACTC-3'		
MET10F	5'-GTTTGTAATAGGAAATGAATTTGGG-3'	320	8		MREV10F	5'-TTTTAGTGTATTTTTGATTAAATTTTTTTT-3'	309	3
MET10R	5'-CTAAATTAAACCACACTCCCCATAC-3'				MREV10R	5'-AAATATTATCTCAACTCAAATTCTAACTTC-3'		
MET11F	5'-GATGTATATATATAAATGGTAATATTTTGG-3'	361	3		MREV11F	5'-TTGTTTTAGTAAATAGGTGATTGATTAAGT-3'	262	2
MET11R	5'-AATTTAAATTTCTAATACATAATAAATAAT-3'				MREV11R	5'-CCAATTAAAAAAATAAAATTAAAAAAATTA-3'		
MET12F	5'-TTTATGTGTTTATTATTAATTAAATTAATT-3'	227	7		MREV12F	5'-AATAAGAGAGTTAAAAATTAGTATTTGT-3'	185	3
MET12R	5'-ACCACACTTATATAACCTTATATCTTTAAA-3'				MREV12R	5'-AAAAAATCAATCTAAATAAAAAAAA-3'		
					MREV13F	5'-TGAGTATTATTTTGAAGTTAGTTGGTAGT-3'	276	5
					MREV13R	5'-CCATCAACAAAAACAATTAATCAATT-3'		
					MREV14F	5'-TTGAAGTAGAAAGGAATTAATTGAAGTAAG-3'	239	12
					MREV14R	5'-AATCTTTAAACTAACCAACTATCTTTA-3'		

Deep sequencing was executed on an Ion Torrent sequenator using the IonXpress barcode set and the 316D chip kit, after a DNA library preparation from the equimolarly pooled bisulfite PCR fragments by the NEBNext® Fast DNA Fragmentation & Library Prep Set for Ion Torrent (New England BioLabs, Ipswich, MA, USA). For data procession the 2.2 Torrentsuite software was used. To gain quality data, reads under 15 average Phred score were omitted. CLC Genomics Workbench 5.5 was used for data analysis. High confidence of the evaluation was ensured by excluding short reads (<20 nucleotide) and setting Length fraction and Similarity fraction parameters to 0.9.

### Creation of the mutant viruses

Seven mutant viruses (3 single M1, M2, M3, and three double M12, M23, M13 and one triple M123) were rescued containing extra CpGs. To introduce CpG mutations into the *Nco*I-*Sac*I (3473-3935) fragments of NADL-2 strain by joining PCR, three pairs of overlapping mutational primers (M1-3F and M1-3R), two external (mut_exF, mut_exR) and two internal (mut_intF, mut_intR) primers were designed (Table 2). For the upstream fragments of the joining PCRs mut_exF and M1-3R primers were utilized, for the downstream fragments PCR mut_exR and M1-3F primers. For single mutants the pN2 infectious clone of the NADL-2 strain [35], for double mutants the clone of the M1 and M2 mutant viruses, while for the triple mutant the clone of M12 served as templates. The PCR reactions included 5 µl of 5X HF Buffer, 0,5 µl of 10 mM dNTP, 1 µl of each overlapping and external primers (15 pmol/µl) of each different mutants, 0,2 µl of 2U/µl Phusion Hot Start II DNA Polymerase (Thermo Fisher Scientific), 0,05 ng template DNA and distilled water to a final volume of 25 µl. Amplifications started by initial denaturation for 5 min at 95 °C and followed by 30 cycles at 98 °C for 10 s, 65 °C for 15 s, and 72 °C for 20 s. Joining PCRs were carried out by mut_intF and mut_intR primers using Hot Start II DNA Polymerase and 2 ng of the appropriate mutant downstream and upstream fragments. DNA amplifications started by initial denaturation for 5 min at 95 °C, followed by 30 cycles at 98 °C for 10 s, 60 °C for 15 s, and 72 °C for 20 s. The amplified 1005 nucleotide-long products were digested with *Nco*I and *Sac*I (New England BioLabs) restriction enzymes and cloned into the same sites of the pN2 infectious clone. Mutant sequences were deposited into GenBank under the accession numbers: KF913345-KF913351.

**Table 2:** Primers for creating mutants with extra CpGs.

**Primer name**	**Sequence**	**Number of extra CpGs**
M1F	5'-ACCCGCCGCTAACACAAGAAAAGGTTATCACCAAACAATTAATAATAGCTACACCGAAGCA ACCGCAATTAGGCCCGCTCAGGTCGGATATAATACACCA-3'	9
M1R	5'-ACCTTTTCTTGTGTTAGCGGCGGGTAGTGTTCCGGGGTGTTGGTCTCCTTCGGTGGTAGG TTCGGTTAGTAGTTTTGGA-3'	
M2F	5'-GTTCTACACAATCGAAAACGCCGTACCAATTCATCTTCTAAGAACCGGCGACGAATTCTCC ACCGGAATATATCACTTTGACACAAA-3'	10
M2R	5'-GGTACGGCGTTTTCGATTGTGTAGAACATAATGTCGCTGTGTAGTCCGGTTTGTATTGAGT CGGTTATTTGTTGTGATTGTCCAGTGTATG-3'	
M3F	5'-TGAATTTCGAATACTCCAACGGCGGACCATTTCTAACTCCTATCGTACCAACCGCCGACAC ACAATATAACGACGACGAACCAAACGGTGCTATAAGATTTA-3'	13
M3R	5'-TGGTCCGCCGTTGGAGTATTCGAAATTCATGTATGGTGTATTATATCCGACCTGAGCGGGC CTAATTGCGGTTGCTTCTGTGTAGCTATT-3'	
mut_exF	5'-ACAGAATCAGCAACCTCACCACCAA-3'	
mut_exR	5'-TGTTCCTTTCCACCAAAAGTTTGAA-3'	
mut_intF	5'-TAGACACCAATAACACACTTCCATA-3'	
mut_intR	5'-TGAGGAGAGTCAGCATTGAA-3'	

### Transfection, viral stocks titration and quantification

To rescue the mutant viruses the infectious clones were transfected into PT cells by Turbofect (Thermo Fisher Scientific) reagent according to the supplier’s recommendations. After 48 hours 50 µl culture media was transferred from the transfected cells to a 24-well plate seeded with PT cells and the viruses were let to multiply for 96 hours. This was followed by the inoculation of semi confluent PT cells with 0.5 ml of viral supernatants on 75 cm^2^ plates. After 96 hours the supernatants were collected and titrated by three parallel, independent dilutions with immunofluorescence detection technique on PT and Cos7 cells as described previously [36]. Shortly: virus samples were serially diluted (10x) and cells on a 96-well plate were infected with 10 µl of the viral dilutions. PT and Cos7 cells were fixed after 20 and 48 hours respectively (to exclude the detection of progeny viruses emerging from the cells used for titering) with 3% formaldehyde and permeabilized with 1% Triton X-100. The 3C9 (CRL-17; ATCC) anti-PPV antibody and Alexa Flour^®^ 488 donkey anti-mouse IgG (Life Technologies Carlsbad, CA, USA) as secondary antibody were used for visualization of the infected nuclei (IN). Titer was calculated by multiplying the number of IN by the dilution factors and values are given in fluorescent nuclei count (FNC)/ml.

For qPCR quantification of the viral production initiated by differently methylated DNAs the transfected cells were washed three times to ensure minimal contamination of the viral stocks by plasmid originated viral DNA.

To monitor sequence stability viral stocks were passaged 10 times on a 24-well plate containing PT cells transferring 5 µl of the virus containing supernatant after 48 hours to freshly seeded cells covered by 2 ml medium. After the last passages new stocks were prepared on 75 cm^2^ plates as it is described above. Following DNA extraction, the mutated regions were amplified by mut_intF, mut_intR primers (Table 2) and sequenced.

For DNA quantification qPCR were performed using the earlier described VPS2 (5’-CAATACTGCACCTGTATTTCCAAATGG-3’) and VPAS2 (5’-AAAATTTTATTGTTTTTTGGGGATAATTGG-3’) primers [37].

The reactions included 2,5 µl of 10X DreamTaq Buffer, 0,5 µl of dNTP mix, 1 µl of each 15 pmol/µl primers, 0,2 µl of DreamTaq DNA Polymerase, 1,25 µl of 20X EvaGreenTM (Biotium, Hayward, CA, USA), 1 µl DNA template and sterile distilled water to a final volume of 25 µl. The DNA amplifications were carried out by initial denaturation for 5 min at 95 °C, followed by 40 cycles at 95 °C for 20 s, 55 °C for 20 s, and 72 °C for 50 s.

### Preparation of bacterially methylated and non-methylated DNA for transfection

To get unmethylated viral genome, a PCR was performed using pN2 as template and N2F (5’-GGGTTATTGTCTCATGAGCGGATACATA-3’) and N2R (5’-CAATTTCACACAGG AAACAGCTATGACC-3’) primers. The PCR reaction included 20 µl of 5X GC Buffer, 2 µl of 10 mM dNTP, 6 µl DMSO 0.6 µl of each 100 pmol/µl primers, 1 µl of 2U/µl Phusion Hot Start II DNA Polymerase (Thermo Fisher Scientific) and distilled water to a final volume of 100 µl. The DNA amplification started by initial denaturation for 3 min at 98 °C, followed by 25 cycles at 98 °C for 15 s, 66 °C for 20 s, and 72 °C for 4 min 30 s. To obtain bacterially DAM and DCM methylated viral genome the pN2 was digested by *Kpn*I and *Bam*HI restriction enzymes (Thermo Fisher Scientific) which cut the NADL-2 virus from the vector. In each case the viral genomes were isolated from 0.7% agarose gel by Nucleospin Extract II kit (Macherey-Nagel, Dueren, Germany).

### In vitro CpG methylation

In a reaction 3 µg viral DNA was methylated by CpG methylase (Zymo Research, Irvine, CA, USA) according to the manufacturer’s instructions. The reaction was stopped by ethanol precipitation, washed by 70% ethanol, dried and resolved in 20 µl distilled water. The success of hipermethylation was inspected by digestion of an aliquot with methylation sensitive *Ssi*I (Thermo Fisher Scientific) restriction enzyme in 20 µl final volume. 

### Determination of expression levels of DNA methyltransferases in infected and non-infected tissues

The expression levels of porcine DNMT1, DNMT3a, DNMT3b were defined by real-time PCR, and normalized with those of the GADPH gene. RNAs were purified from PPV infected (MOI 3) and mock infected PT cells grown on 75 cm^2^ flask 24 hours postinfection (HPI) by RiboZol^TM^ RNA Extraction Reagent (Amresco, Solon, OH, USA) according to the manufacturers’ instructions. The RNA was dissolved in 15 µl DEPC-treated water. The first strand of cDNA and the amplification were created using One-Step RT-PCR Kit (Qiagen). The reactions included 5 µl of 5X QIAGEN OneStep RT-PCR Buffer, 1 µl of dNTP mix, 1 µl of each 15 pmol/µl primers (Table 3), 1 µl of QIAGEN OneStep RT-PCR Enzyme mix, 1,25 µl of 20X EvaGreen^TM^, 1 µl RNA template and RNase-free water to a final volume of 25 µl. The reaction started with reverse transcription for 30 min at 50 °C and initial PCR activation for 15 min at 95 °C, followed by 35 cycles at 95 °C for 30 sec, 55 °C for 30 sec, and 72 °C for 40 sec, and 72 °C for 5 min. 

**Table 3:** Porcine specific primers used for qRT-PCR.

**Primer name**	**Sequence**	**Product size (bp)**	**Accession number**
DNMT1_F	5’-TCGAACCAAAACGGCAGTAGT-3’	215	NM_001032355
DNMT1_R	5’-CGGTCAGTTTGTGTTGGAGAAG-3’		
DNMT3a_F	5’- CTGAGAAGCCCAAGGTCAAG-3’	238	NM_001097437
DNMT3a_R	5’-CAGCAGATGGTGCAGTAGGA-3’		
DNMT3b_F	5’-AACCCAACAAAGCAACCAG-3’	275	CN_156332
DNMT3b_R	5’-CCGACCACAGGATAAACAG-3’		
GADPH_F	5'-TCGGAGTGAACGGATTTG-3'	151	AF_017079
GADPH_R	5'-CCTGGAAGATGGTGATGG-3'		

### Immunofluorescence and Western blot detection of DNMTs

Western blot detection of the DNMT proteins were carried out by using DNMT1 (H-300) and DNMT3a/b (C-15) primary antibodies (Santa Cruz Biotechnology Inc., Heidelberg, Germany), horseradish peroxidase conjugated swine anti-rabbit (DAKO, Glostrup, Denmark) and rabbit anti-goat (Southern Biotech, Birmingham, AL, USA) secondary antibodies in a 50, 100, 500 and 8000-fold dilutions respectively. For loading control anti-alpha tubulin antibody (Developmental Studies Hybridoma Bank, Iowa City, IA, USA) were used together with peroxidase conjugated rabbit anti-mouse IgG (Sigma-Aldrich, St. Louis, MO, USA).

The peroxidase was revealed using the TMB cholorimetric substrate (MIKROGEN, Neuried, Germany) according to the supplier’s recommendations. Bands were quantified with the ImageJ programs [38]. 

For immunofluorescence detection DNMT1 (H-300) and CF488A labeled goat anti-rabbit were used in 50 and 300-fold dilutions respectively and the samples were examined with a Zeiss Axio Observer D1 inverse fluorescence research microscope.

## Results

### GC and CpG content of Parvoviruses

**Figure 1:**
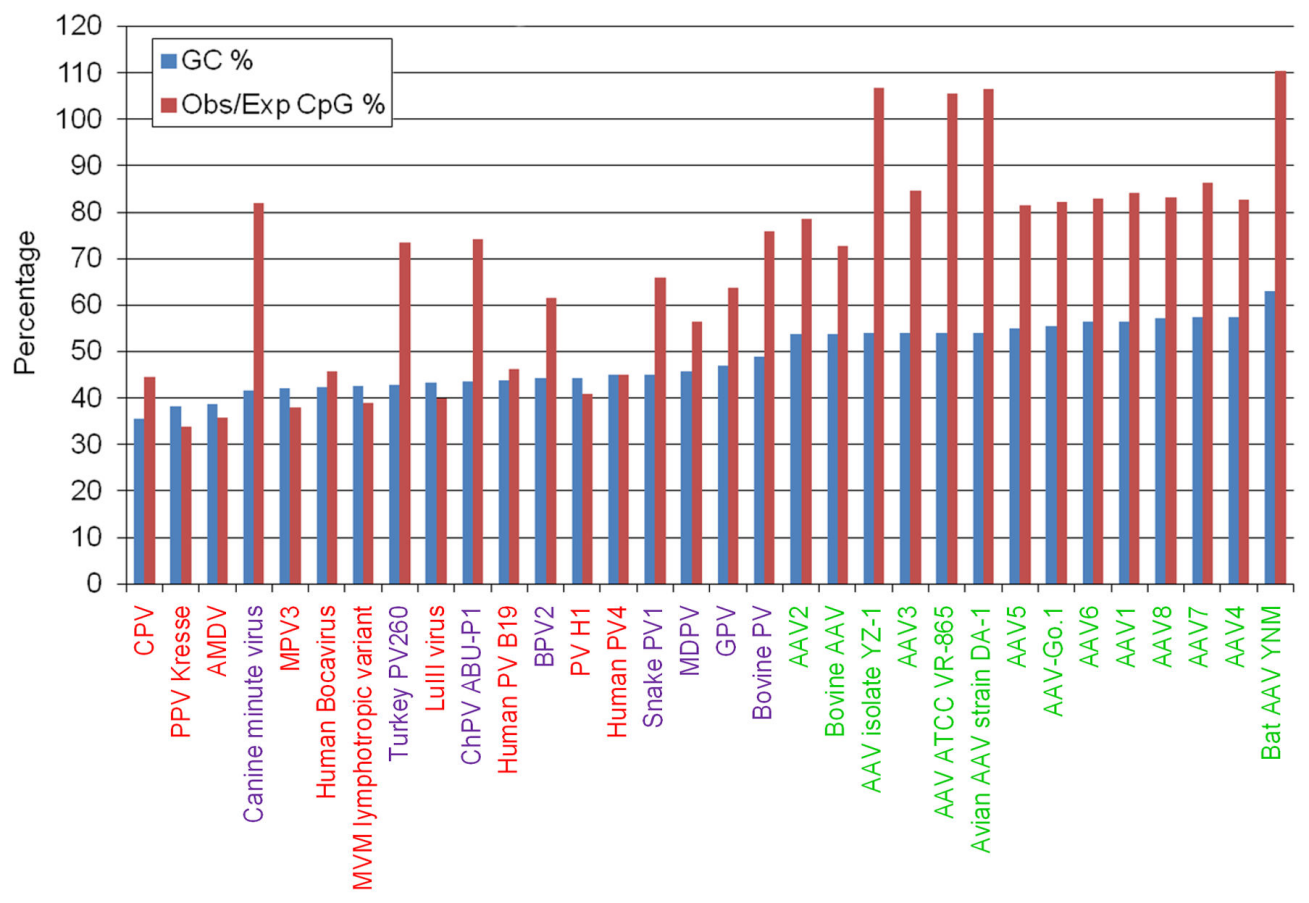
GC content and observed/expected CpG ratio in parvoviral genomes. Values are shown in percentage; viruses are listed by increasing GC content. Group I, Group II and Group III parvoviruses are indicated by red, violet and green colors respectively.

To better understand common and distinguishing features of PPV genome organization among parvoviruses the GC content and CpG density of 32 parvoviruses from the *Parvovirinae* subfamily were calculated and compared. The GC content of parvoviral genomes scales between 35% and 63%. In general, it can be stated that self-replicating parvoviruses have AT-rich genomes (GC content < 50%), while most of the adeno-associated viruses have GC-rich genomes (GC content > 50%) (Figure 1). 

In self-replicating viruses the observed/expected CpG ratio (oCpGr) is variable: it can take very low values (like in the case of PPV), or high values (as it can be seen in the CaMiV genome). Dependoviruses are more uniform regarding CpG content: in each case the oCpGr values stay above 60% with the exception of MDPV (which is an autonomous member of the Dependovirus genus) and can reach more than 100%.

Considering the GC content and the oCpGr values, three major categories can be distinguished among the members of *Parvovirinae* subfamily: parvoviruses with low GC content (< 50%) and low oCpGr values (< 50%) (Group I), viruses with low GC content (< 50%) and high oCpGr values (> 50%) (Group II) and viruses with high GC (> 50%) content and high oCpGr (> 50%) values (Group III). A characteristic taxonomical distribution can be recognized among these three groups. All known members of Amdovirus, Erythrovirus and Parvovirus (PPV among them) genera together with several members of the Bocavirus genus (like the highly CpG depleted human bocavirus and porcine bocavirus) belong to Group I. Other members of the Bocavirus genus (e.g. the CpG-rich BPV and CaMiV) together with the unclassified chicken and turkey parvoviruses are members of Group II. Most viruses of the Dependovirus genus belong to Group III.

### CpG distribution in PPV and in the coding regions of parvoviruses

**Figure 2:**
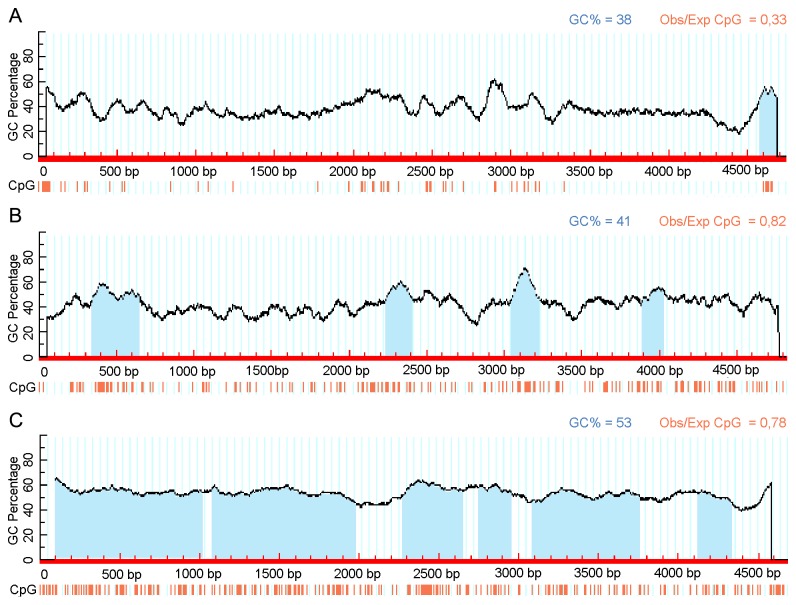
CpG plot of three parvoviral genomes. Red vertical bars represent CpG sites in the genomes, light blue shading indicates CpG islands (parameters: window 100, observed/expected CpG minimum 0.6, GC% minimum 50). A, PPV Kresse strain; B, Canine minute virus; C, AAV2 as representatives of Group I, Group II and Group III parvoviruses respectively. Figure was generated by MethPrimer program [34].

Not only the number of CpGs but their distribution in the genome is different among parvoviruses. Very few CpG islands can be found in members of the first group and usually they are restricted to the terminal regions, while several potential CpG islands can be plotted in every member of the latter two groups scattered throughout the genome including both the coding and non-coding regions (Figure 2).

**Figure 3:**
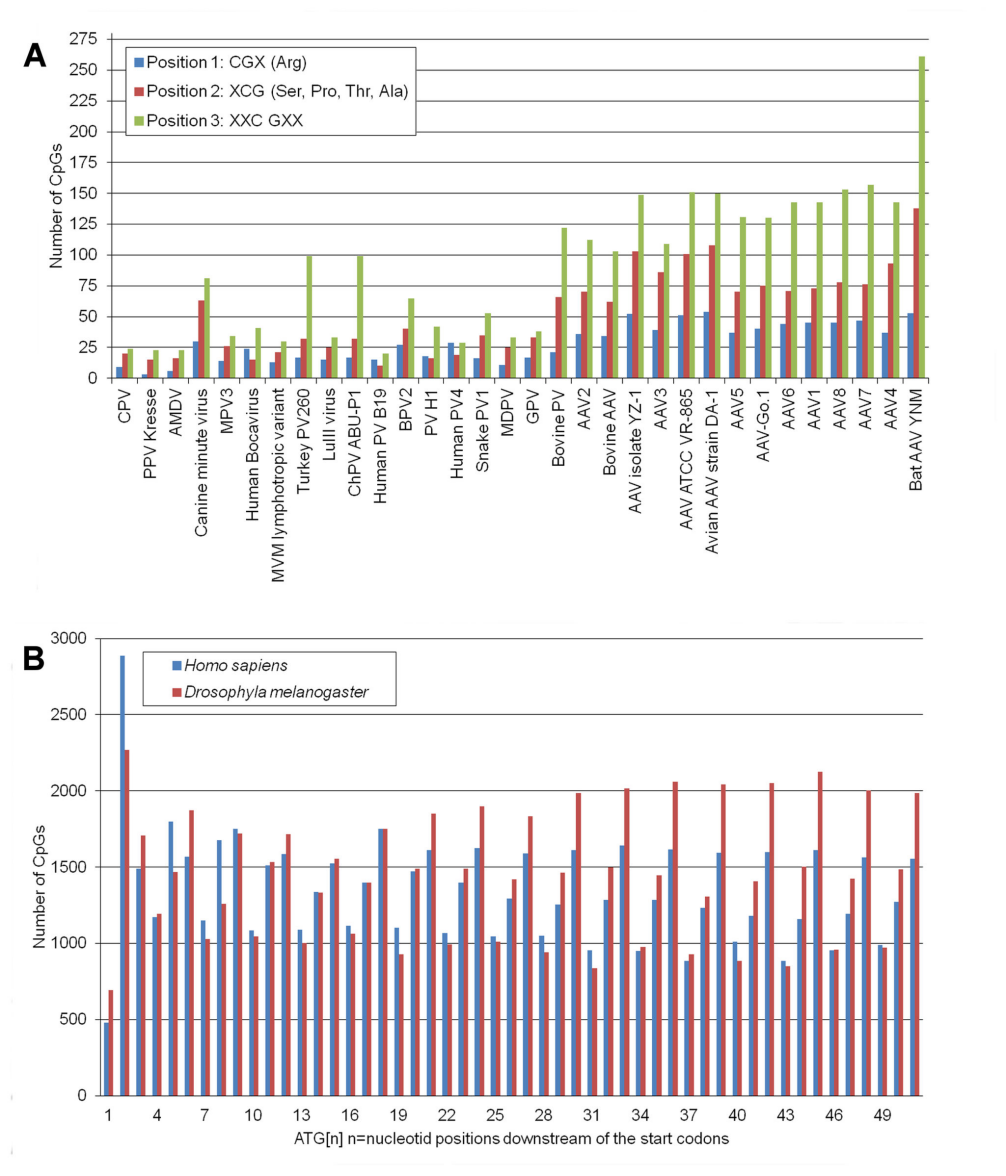
CpG distribution in the coding positions of different organisms. A, Total number of CpGs in the main coding ORFs (NS and VP) of parvoviral genomes. B, Number of CpGs in the different positions of the mRNAs of the human and drosophila proteomes. Numbers on the X axis indicate distance from the start codon of the proteins; Number of CGX (Arg codons) are indicated in 1,4,7,10… positions, numbers of XCG codons (Ser, Pro, Thr, Ala) are indicated in 2,5,8,11… positions and numbers of CGs in consecutive codons XXC GXX are indicated in 3,6,9,12… positions.

The NADL-2 strain of PPV contains 60 CpGs 13 in the left 7 in the right non coding regions and 40 in the protein coding sequences. The distribution of CpGs in the coding frames is not random. Only three CpGs can be found in the first coding position (CGX) in the Arginine codons, 13 in the second position (XCG) (in serine, proline, threonine and alanine codons) and 24 in the third position (XXC GXX) affecting two amino acid codons (Figure 3a). Similar ascendant tendency of CpG distribution can be observed by position in the majority of the parvoviruses but also in the viral hosts, for example in *Homo sapiens* (Figure 3b). Since the distribution is independent of the absolute number of the CpGs in the viral genomes and similar distribution can be detected in eukaryotic organisms, which do not have CpG methylation (e.g. *Drosophila melanogaster*) [39], it is more probable that the particular pattern of CpG distribution is rather the effect of coding bias and coding preference than some unknown evolutionary effect of the methylation machinery of the different organisms. 

### Biological effects of additional CpGs in the PPV genome

**Figure 4:**
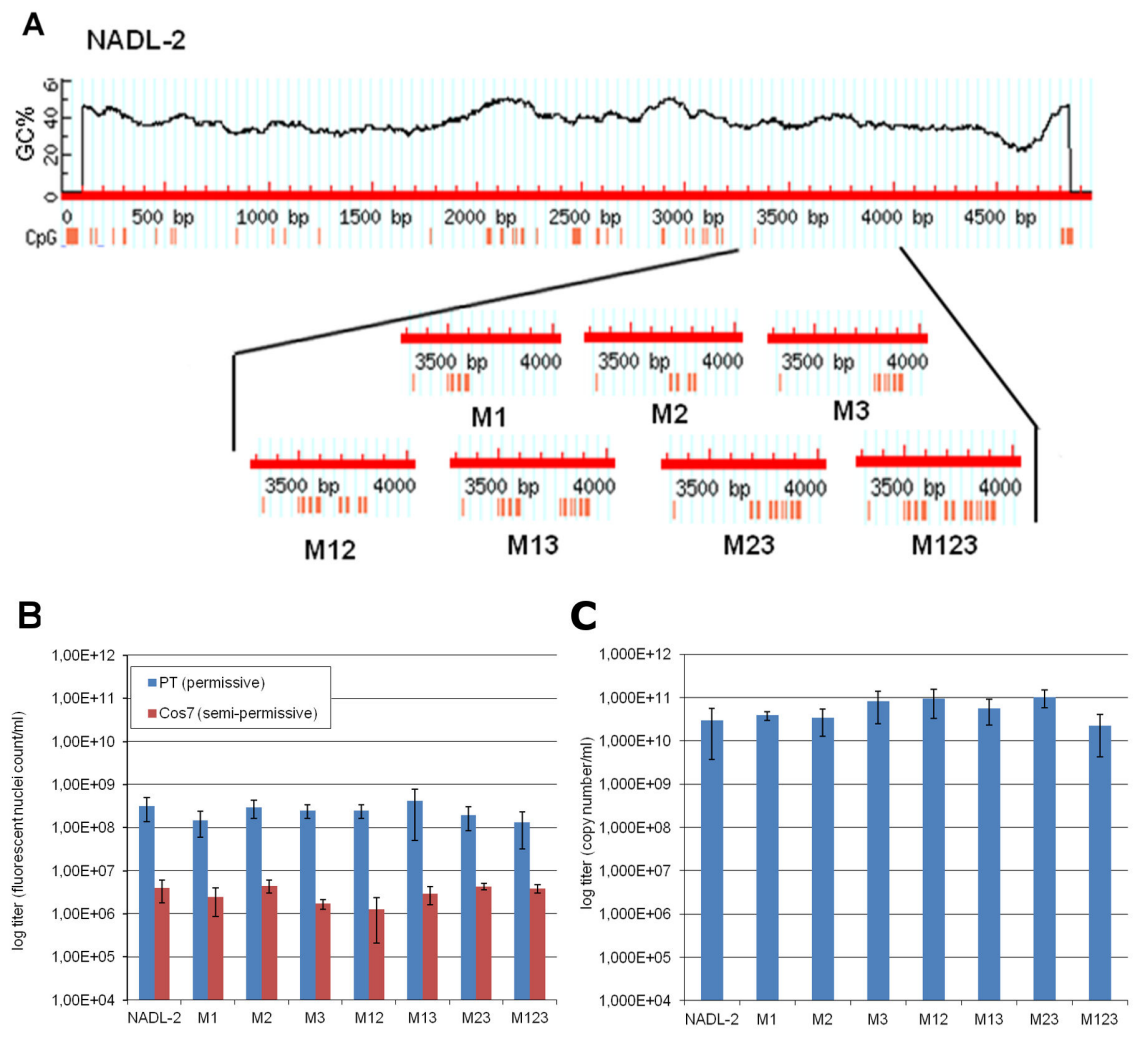
Titration of the mutant PPVs with extra CpGs. A, Schematic representation of the novel CpG sites in the mutant viruses. Red vertical bars symbolize the CpGs in the genomes. B, Titer of the mutant stocks on PT and Cos7 cells. Infected cells were detected by immunofluorescence labeling at 20 and 48 hours PI fixation, respectively. C, Titer of the mutant stocks after 10 additional passages on PT cells. Quantification was carried out by qPCR. Vertical bars indicate the standard deviation in each case.

The small number of CpGs in the PPV genome implies an evolutionary pressure against this dinucleotide. To study the biological effects of the elevated CpG ratio, seven mutants were created in which new CpG sites were inserted into three CpG free regions of the VP2 gene by site-specific silent mutations of the pN2 infectious clone. The number of the new CpG sites in the mutants ranged between 9 and 29 (Figure 4a). The mutant clones were transfected into PT cells and the supernatants of the transfected cells were collected 48 hour post transfection and used to infect PT cells. The presence of PPV was monitored by IF using 3C9 monoclonal antibodies. The transfection of all mutant infectious clones resulted viable virus and a stock solution was prepared from each and titrated on permissive PT cells and semi-permissive Cos7 cells. There was no significant difference in the infectivity of the mutant and the wild type viral stocks indicating that the extra CpG sites have no significant biological effects (p= 0.557 at PT cells and p= 0.0727 at Cos7 cells by ANOVA) either on viral production or viral growth *in vitro* (Figure 4b). To monitor the stability of the mutant viruses 10 additional passages were carried out on PT cells and new stock solutions were prepared. The titers of the stocks were quantified by qPCR (Figure 4c) and again there were no significant differences (p= 0.0974 by ANOVA) in the titer among the different mutants and the wild type virus. Sequencing of the mutant viruses from the different mutant stocks confirmed that no new or back mutations are present on the viruses and the introduced mutations are stable, which indicates lack of selective disadvantage of the additional CpGs in tissue culture.

### Methylation status of PPV

To establish the methylation status of PPV genome, the bisulfite conversion based PCR protocol was used, which allows the independent analysis of the methylation of the negative and the positive strands. To cover all CpG sites on the full genome two sets of PCR primers (13 pairs for the negative strand and 11 pairs for the positive strand) were planned. First, the encapsidated negative strand was analyzed from virions originated from permissive (PT) 20 HPI semi-permissive (Cos7) cells 96 HPI and aborted pig embryos. In each case the investigated DNAs were highly hypomethylated (Table 4, Table S2). PPV encapsidates only negative strand, and positive strand can be found almost exclusively only in the replicative form PPV DNA [40]. To exclude that the hypomethylation pattern of the viral DNA would be the result of specific encapsidation of the unmethylated DNA, the methylation pattern of the cellular PPV DNA purified from PPV infected PT cells (20 HPI) was also determined (Table 4). Similarly to the encapsidated negative strand, the positive strand proved to be hypomethylated, suggesting that PPV DNA remains hypomethylated during the entire life cycle of the virus including replication and packaging.

**Table 4:** Methylation of the CpG sites in PT cells 20 hours post infection.

	**Positive strand**				**Negative strand**		
**CpG site**	**Position**	**Clones sequenced**	**Methylation**		**Position**	**Clones sequenced**	**Methylation**
1	28	6	0		29	6	0
2	38	6	0		39	6	0
3	46	6	0		47	6	0
4	48	6	0		49	6	0
5	50	6	0		51	6	0
6	55	6	0		56	6	0
7	57	6	0		58	6	0
8	59	6	0		60	6	0
9	68	6	0		69	6	0
10	79	6	0		80	6	0
11	147	6	0		148	12	0
12	168	6	0		169	12	0
13	249	4	0		250	6	0
14	299	4	0		300	6	0
15	314	4	0		315	6	0
16	455	8	0		456	6	0
17	531	4	0		532	6	0
18	547	4	0		548	6	0
19	845	3	0		846	6	0
20	1017	3	0		1018	11	0
21	1079	7	0		1080	5	0
22	1239	4	0		1240	5	0
23	1776	4	0		1777	6	0
24	2051	6	0		2052	5	0
25	2057	6	0		2058	5	0
26	2070	6	0		2071	5	1
27	2120	6	0		2121	4	0
28	2127	6	0		2128	4	0
29	2174	6	0		2175	4	0
30	2191	6	0		2192	4	0
31	2213	6	0		2214	4	0
32	2226	6	0		2227	4	0
33	2291	6	0		2292	4	0
34	2464	6	0		2465	6	0
35	2467	6	0		2468	6	0
36	2482	6	0		2483	6	0
37	2485	6	0		2486	6	0
38	2494	6	0		2495	6	0
39	2575	6	1		2576	12	0
40	2587	6	0		2588	12	0
41	2624	6	0		2625	6	0
42	2694	6	0		2695	6	0
43	2697	6	0		2698	6	0
44	2893	3	0		2894	6	0
45	2896	3	0		2897	6	0
46	2902	3	0		2903	6	0
47	3004	3	0		3005	6	0
48	3040	3	0		3041	6	0
49	3087	3	0		3088	4	0
50	3106	3	0		3107	4	1
51	3154	7	0		3155	4	0
52	3180	4	0		3181	4	0
53	3334	4	0		3335	4	0
54	4940	6	0		4941	6	0
55	4950	6	0		4951	6	0
56	4962	6	0		4963	6	1
57	4971	6	1		4972	6	0
58	4983	6	0		4984	6	0
59	4987	6	0		4988	6	0
60	4990	6	0		4991	6	0

**Figure 5:**
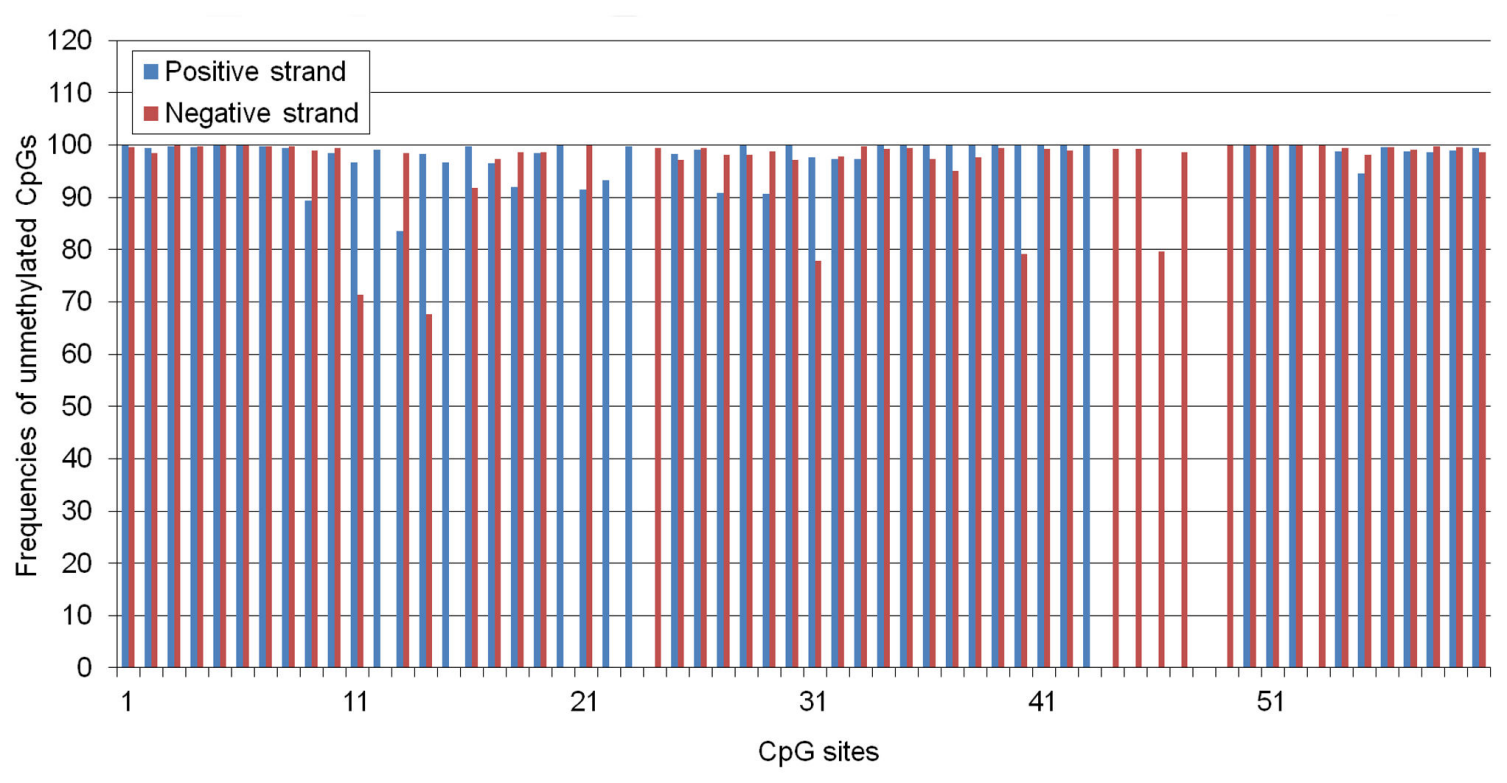
Deep sequencing of the bisulfite treated PPV genome. Vertical bars label the position of the CpGs in the PPV genome. Height of the bars represents the frequency of unmethylated CpGs. Coverage of the CpG sites having more than 8% methylation rate was between 47 and 13694.

1-2 percentage points of the CpG sites on the cloned bisulfite treated PPV fragments remained resistant against C to T conversion indicating a rare occurrence of methylation on the PPV DNA. To gain more accurate data about the methylation level, the bisulfite treated PCR fragments were deep sequenced. Around 168000 PCR fragments were analyzed with 0-22619 coverage of the CpG sites. Most of the CpG sites had more than 92% conversion frequency while around 10% of the CpG sites had less than 92% conversion rate indicating that low level CpG methylation occurs on replicating PPV DNA (Figure 5). Most probably there is no immediate effect of such a low level methylation on PPV replication, however, even low level of methylation can drive the purge of CpGs from the PPV genome during a long period of time, since methylated cytosines are mutational hotspots [41,42].

**Figure 6:**
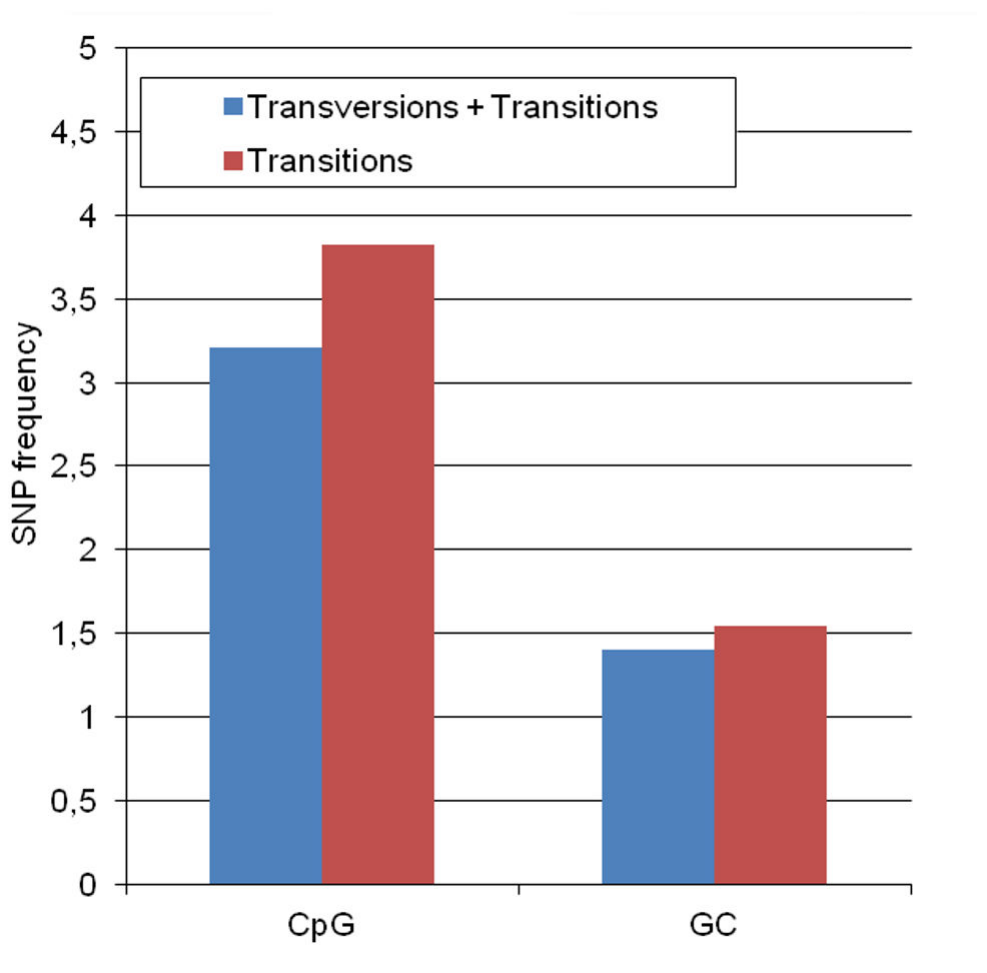
SNP frequency of the CpG and GC sites in the PPV genome. The frequency was calculated by dividing the rate of SNPs in the CpG and GC sites with the rate of SNPs in the total number of Gs and Cs in the genome.

In fact, analysis of single nucleotide polymorphism (SNPs) of the PPV genome based on the available sequences of the DNA databank support the high mutability of CpG sites in the PPV genome. On the investigated coding region, the ratio of SNPs in the CpG sites (17 mutations, 38 CpG sites) significantly exceeds the ratio of SNPs in the total number of G and C nucleotides (113 mutations, 1619 G+C nucleotides) (p< 2.2x10^-16^ by Pearson's Chi-square test) or in the GC sites (29 mutations, 148 GC sites) (p= 0.0014 by Pearson's Chi-square test). Even a higher difference (3,82) can be observed calculating the transition rates (p= 5.413x10^-13^) by Pearson's Chi-square test) (14 C→T and G→A in CpG sites versus 78 in G and C nucleotides of the genome) (Figure 6).

These data, together with our observation that extra CpG sites in PPV do not interfere with PPV replication, raise the possibility that not only natural selection but mutational pressure might contribute significantly to the low level balance of the CpG sites in the PPV genome.

### The effect of methylation on PPV replication

**Figure 7:**
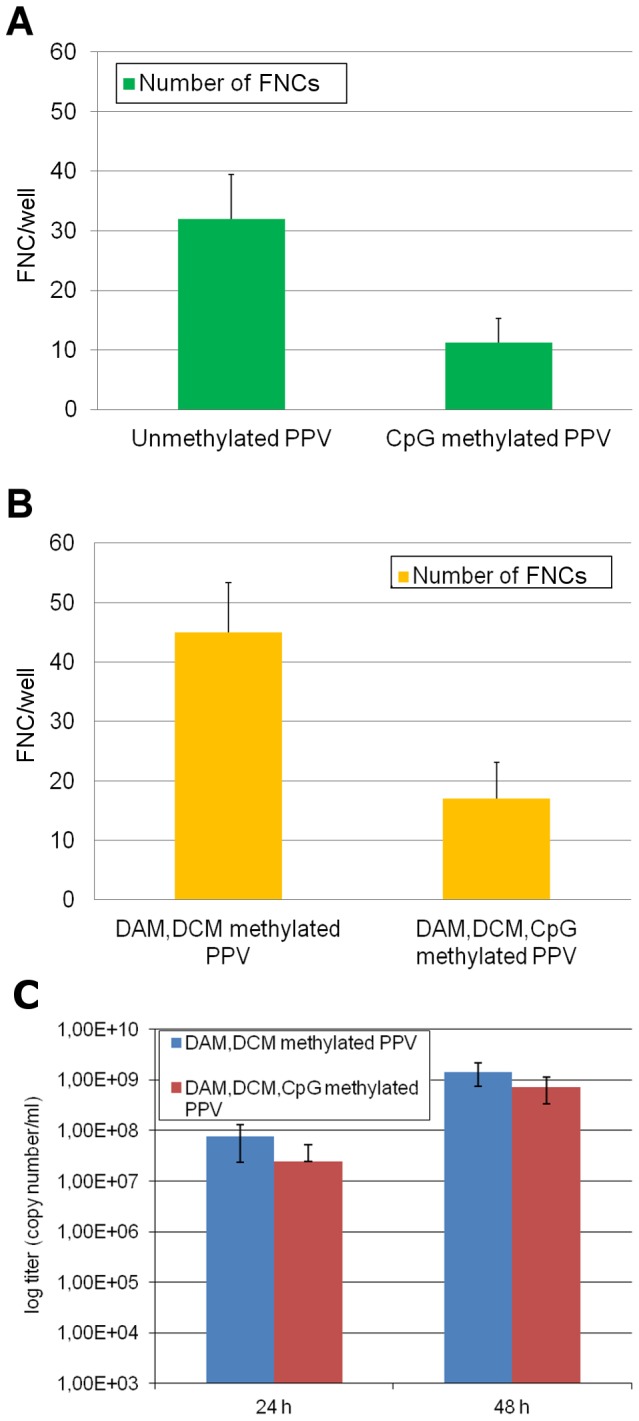
Initiation of the replication by differentially methylated PPV DNAs. A, and B, PT cells on 96 well plate were transfected by 0.2 µg differentially methylated DNA and PPV infected cells were detected by immunofluorescence 24 hour post-transfection. The fluorescent nuclei count (FNC) in one well is indicated by columns. C, Number of viral genomes in the supernatant of differentially methylated DNA transfected cells 24 and 48 hours post-transfection. Vertical bars indicate standard deviation in each case.

To investigate the effect of CpG methylation on the PPV replication, PPV genomes were in vitro methylated, transfected and their replication initiation capability was compared to that of the bacterially cloned (DAM DCM methylated) and PCR amplified (non-methylated) PPV genomes. In vitro methylation was executed by M.SssI CpG methylase and in each case almost complete CpG methylation (>95%) of the PPV genomes could be achieved, shown by the digestion of the treated DNAs with the *Ssi*I methylation sensitive restriction endonuclease (data not shown). The CpG methylated DNAs and their non CpG methylated counterparts were transfected into PT cells and their virus replication initiation capability was monitored by IF assay at 24 hours post-transfection using 3C9 PPV specific monoclonal antibody. In each case the CpG methylated DNA induced around 62% less viral infection in PT cells than the non CpG methylated control DNAs, indicating that CpG methylation has a relatively modest inhibitory effect on PPV replication (Figure 7a-b). This result was confirmed by qPCR analysis of the supernatant of the transfected cells (Figure 7c). Interestingly, non-methylated PPV dsDNA (PCR amplified) was less effective to initiate viral replication than the bacterially DAM/DCM methylated dsDNA. 

The progeny viruses from CpG methylated PPV DNA transfected cells were collected, and the methylation pattern of their DNA was examined. The *in vitro* hypermethylated status of the transfected PPV genome could not be detected on the investigated fragments of the genome of the progeny viruses, they even proved to be hypomethylated, similarly to the genome of the native virus (Table S2d).

To gather additional evidence on the sustainability of the hypomethylated status of the PPV genome, the clone of the M123 mutant was also transfected into PT cells and the methylation pattern of the progeny virus was also examined. The 29 newly introduced CpG sites also remained hypomethylated similarly to the “wild type” CpG sites of the native virus (Table S2e). 

These experiments strongly suggest that beside the maintenance DNMT1 the *de novo* DNMT3a and DNMT3b methylases cannot methylate replicating PPV DNA effectively either, and hypomethylation is not restricted to the existing CpG sites: it is a generalized process which extends to the full PPV genome.

### Influence of the PPV infection on the transcription, translation, and cellular distribution of DNMT proteins

**Figure 8:**
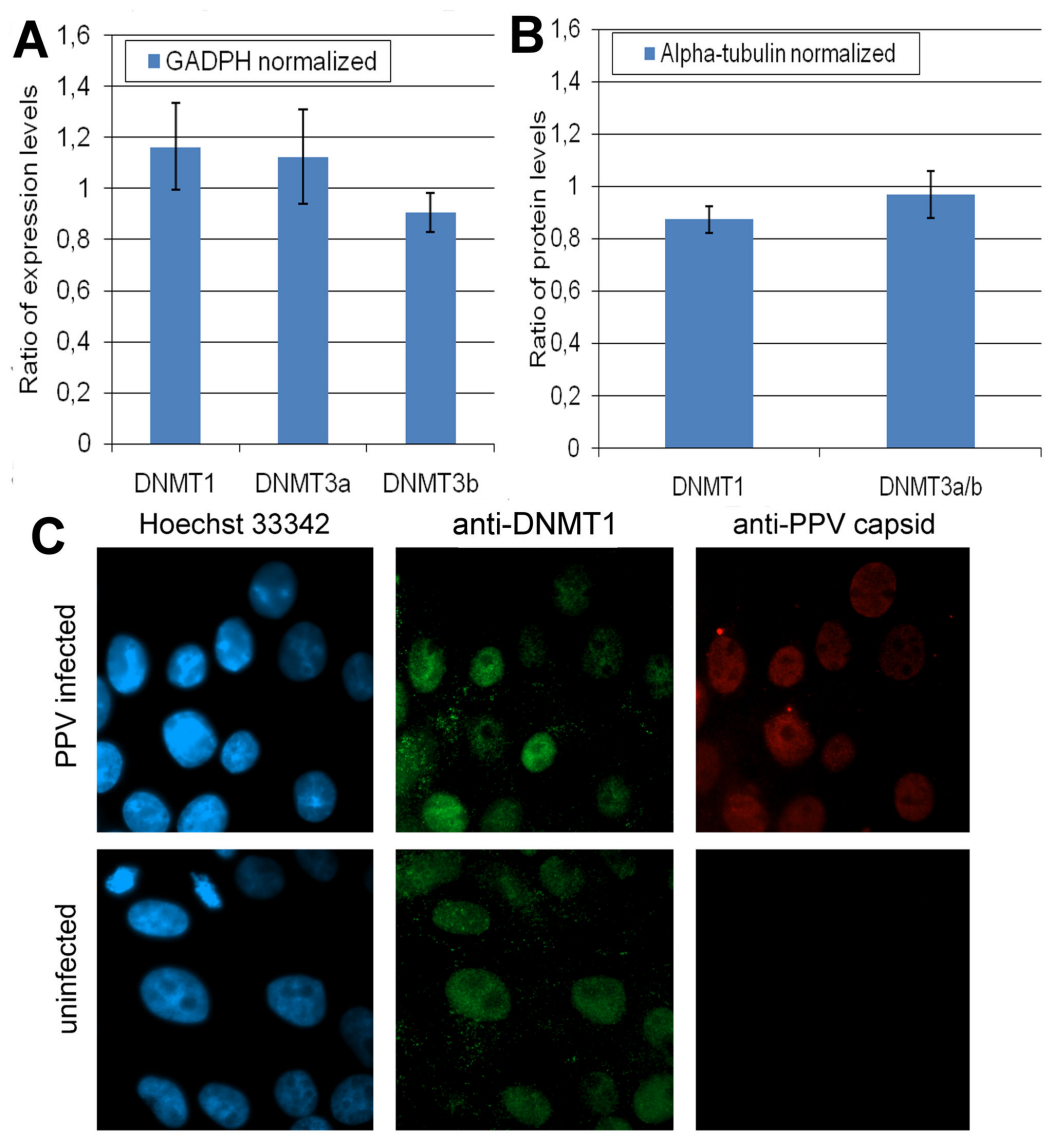
Influence of the PPV infection on the expression of DNMTs. A, Ratio of the mRNA levels of DNMTs in PPV infected/uninfected PT cells. Level of expressions were normalized with GADPH. B, Ratio of the alpha-tubulin normalized DNMT1 and DNMT3a/b protein levels in PPV infected/uninfected PT cells. C, Localization of DNMT1 in infected and uninfected PT cells. Nuclear staining by Hoechst 33342 is shown in blue, DNMT1 and PPV capsid proteins were visualized by indirect immunofluorescence methods and they are presented in green and red, respectively.

Hypomethylation of PPV DNA must involve a decreased activity of the cellular DNMTs on PPV DNA and/or a weak susceptibility of the PPV DNA to their action. Absence, inhibition or elimination of DNMTs in infected cells, different compartmentalization of methylases and viral DNA, or the unability of methylases to recognize PPV DNA as substrate singularly or synergistically can lead to hypomethylation of the viral genome. To study the direct reasons behind the hypomethylated status of PPV DNA we have investigated the transcription, translation and subcellular distribution of DNMTs in infected and non-infected host cells. Quantitative measurement of the mRNAs of DNMT1 DNMT3a and DNMT3b by real-time qRT-PCR reveals that the mRNAs of all the three major form of DNMTs are present in PT cells and PPV infection does not change their levels significantly (Figure 8a). As demonstrated by Western-blot and immunofluorescence experiments, the translation level of the DNMT proteins is not affected significantly and localization of the DNMT1 is not changed either by PPV infection in PT cells (Figure 8b-c). 

The effect of overexpression of the DNMT3a on PPV methylation was also investigated. To ensure the co-expression of the PPV genome and the DNMT3a protein, the pN2 and the human DNMT3a expressing pcDNA3/Myc-DNMT3A [43,44] plasmid were co-transfected into PT cells. Co-transfection of the two plasmids resulted around three times less infectious foci (data not shown) than the cotransfection of the pN2 and the pDSREDmonomer-N1 plasmid which expressed the dsRED protein as a control, indicating an inhibitory effect of DNMT3a on PPV replication. Overexpression of DNMTs has been shown to change the pattern of gene expression in cells and influence cell cycle [45,46]. So it cannot be excluded that the viral inhibition of the overexpressed DNMT3a is rather due to an indirect effect on the host cell regulation than to direct methylase activity on the viral DNA. Especially because bisulfite sequencing of the rescued viruses, emerged from pcDNA3/Myc-DNMT3A and pN2 co-transfection, revealed a hypomethylated status of the viral DNA (Table S2f), demonstrating that DNMT3a, even present in excess, cannot methylate effectively replicating PPV DNA.

## Discussion

Investigation of the GC and CpG contents of 32 parvoviral genomes revealed three distinct groups. Dependoviruses represent a group with high GC and high oCpGr values. As earlier recognized, the relative CpG-rich sequence of dependoviruses makes them an out-group among similarly sized DNA viruses [9,47]. Just like large DNA viruses, they are much less biased toward CpG dinucleotids than the small DNA viruses. 

The majority of the known autonomously replicating parvoviruses belong to a different group with opposing characteristics manifesting low GC and low oCpGr values. However, based on our findings, several recently described autonomous parvoviruses seem to differ significantly from this group and feature relatively high oCpGr values combined with low GC content. 

This finding somewhat extenuates the conclusion which emerged from the earlier analysis of viral sequences that small DNA viruses – parvoviruses among them – are extremely biased against CpG dinucleotide, and only adeno-associated viruses are exceptions from this rule [9,47]. 

The methylation patterns of a few DNA viruses have been studied in details. The hypomethylated state of the replicating PPV genome fits very well with what was described about replicating adeno [48,49] papilloma and polyoma viruses [50,51,52,53] and reinforces the emerging view that the genome of small DNA viruses remains hypomethylated during replication. One possible explanation for this phenomenon is the association of the viral DNA with host and viral proteins during rapid viral replication and encapsidation which may simply prevent the interactions with DNMTs. However, the lack of (so far unknown) cis signals for de novo methylation or the presence of chromatin insulator sequences [54,55,56] might also contribute to the sustainment of hypomethylation in the PPV genome. This notion is supported by the fact that while some episomal adenoviral constructs become rapidly *de novo* methylated [57], others, like episomal recombinant AAV and even some bacterial plasmid constructs remain hypomethylated for a long time after introduction into the mammalian cells, despite being replication incompetent [58,59]. The inverted terminal repeat (ITR) of AAV is suspected to be an insulator [60] and most probably has a role in keeping AAV recombinant constructs hypomethylated and transcriptionally active [61]. However, whether the ITRs of PPV or any other parvovirus functions similarly remains to be investigated.

Interestingly, complete in vitro CpG methylation of PPV genome has a moderate inhibitory effect (around 62% decrease) on PPV replication initiation. The decrease was found very reproducible in several experiments and independent of the original methylation status of the treated DNA. These experiments indicate a moderate sensitivity of the PPV genome to methylation. This result is somewhat contradictory to human parvovirus B19 studies, since B19 promoter and replication seem to be very sensitive to CpG methylation [62]. However, B19 has a CpG island and 42 CpGs in its terminal 520 bp promoter/enhancer region overlapping with Sp1, Sp3 and viral NS protein binding elements, while PPV has no CpG island and has only 12 CpGs in its 190 bp long P4 promoter/enhancer region and no CpGs at all in its 140-bp-long P40 promoter region. Recent investigations revealed that the relationship between the methylation status and transcriptional activity of a gene is more complicated than it was originally thought [4,63,64] and different promoters react differently to methylation [63,65]. The different sensitivity of the two parvoviruses to methylation can be explained by the different CpG content of their promoters since the methylation of CpG-poor promoters frequently does not preclude transcription [63] while the methylation of CpG island promoters usually suppresses their activity [4,65].

In contrast to viruses (e.g hepatitis B virus, Marek’s disease virus, Kaposi's sarcoma-associated herpesvirus and EBV) from other viral families [66,67,68,69], PPV infection does not influence the mRNA or the protein level of the DNA methylases and does not seem to change the localization of the DNMT3a. So it looks like that replicating PPV DNA is a weak substrate of the host’s DNA methylases and even overexpressed DNMT3a cannot raise PPV methylation level significantly. 

The genome of the majority of the autonomous parvoviruses – PPV among them – similarly to their hosts are highly GC and CpG depleted. It is widely assumed that the main reason behind CpG depletion in small DNA viruses and RNA viruses is natural selection coming from replicative advantage and/or immune escape [9,47]. However, our findings that the introduction of additional CpGs into the PPV genome has no measurable biological effect (no disadvantage),or *in vitro* hypermethylation does not significantly inhibit replication initiation of PPV argue against the replicative advantage of CpG depletion. A recent publication about the failure of members of the Parvoviridae family to elicit TLR 9 activated interferon response in plasmacytoid dendritic cells [26] questions the existence of immunological pressure against CpGs in parvoviral genomes. The ascendant distribution of CpGs by position does not support the presence of immunological pressure against CpGs in parvoviruses either because it would be expected that under such pressure the number of first position CpGs would exceed the number of second or third position CpGs (the degenerate code makes it easy to change the C and G of the CpGs in third and second positions without amino acid changes while point mutations of first position CpGs inevitably come with amino acid changes which could be harmful to the virus).

These data together with our observation that CpG sites are more mutable than GC or C and G sites in the PPV genome suggest that mutational pressure could have a more significant role in the formation of PPV genome than selective forces. 

 In vertebrate genomes CpG is the most mutable dinucleotide and its occurrence is about 20 % of the expected frequency [5,6]. The high mutability of CpGs and its frequent conversion to TG and CA largely attributed to mutational pressure caused by the spontaneous deamination of 5-methylcytosine. The low level methylation of the PPV genome combined with the high mutation rate of parvoviruses [70,71] and the single stranded DNA genome, which is very susceptible for deamination [72,73] may explain the higher mutability of CpGs and their loss from the PPV genome through countless generations. Reinforcing this argument we have to mention that not only the transition but the transversion rates of the CpG sites are a few times higher in mammals [74,75,76] and non-methylated CpGs still have an approximately three times higher overall mutation rate in the human genome than non CpG nucleotides [77]. These observations suggest a deamination-independent intrinsic mutability of CpGs in the mammalian genome, and raise the possibility that mutational pressure originating from the host replicative machinery may have a significant influence to CpG suppression, at least in small DNA viruses which lack their own DNA polymerases. 

Similarly to other organisms, mutational pressure, genetic drift and natural selection together shape the genome of parvoviruses. Our *in vitro* investigations of different CpG mutants of PPV were not able to reveal any significant evolutionary advantage of CpG depletion in viral replication at cellular level. Future *in vivo* experiments and monitoring our extra CpG mutants at organism level may help us clarify how much of the loss of CpGs from the PPV genome is the consequence of selective or other evolutionary forces. 

## Supporting Information

Table S1 **Accession numbers of the viral sequences.** 1a, Accession numbers and abbreviations of the investigated parvoviral sequences.
1b, Accession numbers of the PPV sequences.
(DOC)

Table S2 **Methylation level of the CpG sites in the PPV genome.** 2a, Methylation of the CpG sites in PT cells 8 hours post infection. 2b, Methylation of the CpG sites in Cos 7 cells 96 hours post infection. 2c, Methylation of the CpG sites in PPV originated from aborted pig embryos. 2d, Methylation of the CpG sites in the progeny viruses of the *in* *vitro* methylated PPV genome. 2e, Methylation of the new CpG sites in the M123 mutant PPV. 2f, Methylation of the progeny viruses after overexpressing of DNMT3a in PT cells.
(DOC)
