# Supplementary material for: CpG Distribution and Methylation Pattern in Porcine Parvovirus
Source: PLoS One. 2013 Dec 31;8(12):e85986. doi: 10.1371/journal.pone.0085986 (PMC3877397; doi:10.1371/journal.pone.0085986)
Supplement: Table S1 — Accession numbers of the viral sequences. 1a, Accession numbers and abbreviations of the investigated parvoviral sequences. 1b, Accession numbers of the PPV sequences. (DOC) [file pone.0085986.s001.doc]

**Table S1.**

1a,

| **Name of virus** | **Accession number** | **Abbreviation** |
| --- | --- | --- |
| Canine parvovirus | NC_001539.1 | CPV |
| Porcine parvovirus strain Kresse | U44978.1 | PPV Kresse |
| Aleutian Mink Disease Virus | NC_001662.1 | AMDV |
| Canine minute virus | NC_004442.1 | Canine minute virus |
| Mouse parvovirus 3 | NC_008185.1 | MPV3 |
| Human bocavirus isolate WH | FJ496754.1 | Human Bocavirus |
| Minute virus of mice, a lymphotropic variant of MVM | M12032.1 | MVM lymphotropic variant |
| Turkey parvovirus 260 | GU214706.1 | Turkey PV260 |
| LuIII virus | NC_004713.1 | LuIII virus |
| Chicken parvovirus ABU-P1 | GU214704.1 | ChPV ABU-P1 |
| Human parvovirus B19 | NC_000883.2 | Human PV B19 |
| Bovine parvovirus 2 | NC_006259.1 | BPV2 |
| Parvovirus H1 | NC_001358.1 | PV H1 |
| Human parvovirus 4 | NC_007018.1 | Human PV4 |
| Snake parvovirus 1 | NC_006148.1 | Snake PV1 |
| Muscovy duck parvovirus | NC_006147.2 | MDPV |
| Goose parvovirus | NC_001701.1 | GPV |
| Bovine parvovirus | NC_001540.1 | Bovine PV |
| Adeno-associated virus-2 | NC_001401.2 | AAV2 |
| Bovine adeno-associated virus | NC_005889.1 | Bovine AAV |
| Avian adeno-associated virus isolate YZ-1 | GQ368252.1 | AAV isolate YZ-1 |
| Adeno-associated virus-3 | NC_001729.1 | AAV3 |
| Avian adeno-associated virus ATCC VR-865 | NC_004828.1 | AAV ATCC VR-865 |
| Avian adeno-associated virus strain DA-1 | NC_006263.1 | Avian AAV strain DA-1 |
| Adeno-associated virus 5 | NC_006152.1 | AAV5 |
| Adeno-associated virus-Go.1 | DQ335246.2 | AAV-Go.1 |
| Adeno-associated virus 6 | AF028704.1 | AAV6 |
| Adeno-associated virus-1 | NC_002077.1 | AAV1 |
| Adeno-associated virus-8 | NC_006261.1 | AAV8 |
| Adeno-associated virus-7 | NC_006260.1 | AAV7 |
| Adeno-associated virus-4 | NC_001829.1 | AAV4 |
| Bat adeno-associated virus YNM | GU226971.1 | Bat AAV YNM |

1b,

| **Accession numbers of PPV sequences** | | | |
| --- | --- | --- | --- |
| 1 | GI:1255777 | 35 | GI:332983 |
| 2 | GI:194277764 | 36 | GI:56484937 |
| 3 | GI:385166 | 37 | GI:56484836 |
| 4 | GI:374676766 | 38 | GI:342315708 |
| 5 | GI:332987 | 39 | GI:56484767 |
| 6 | GI:194277770 | 40 | GI:56484866 |
| 7 | GI:308220207 | 41 | GI:56484732 |
| 8 | GI:307827191 | 42 | GI:382976673 |
| 9 | GI:56484797 | 43 | GI:382976697 |
| 10 | GI:307827209 | 44 | GI:382976688 |
| 11 | GI:56484901 | 45 | GI:382976685 |
| 12 | GI:307827161 | 46 | GI:382976679 |
| 13 | GI:307827185 | 47 | GI:382976670 |
| 14 | GI:307827173 | 48 | GI:124021378 |
| 15 | GI:307827179 | 49 | GI:382976691 |
| 16 | GI:307827215 | 50 | GI:382976682 |
| 17 | GI:307827221 | 51 | GI:382976676 |
| 18 | GI:307827227 | 52 | GI:382976737 |
| 19 | GI:307827203 | 53 | GI:382976709 |
| 20 | GI:307827167 | 54 | GI:382976706 |
| 21 | GI:46404506 | 55 | GI:382976700 |
| 22 | GI:110617785 | 56 | GI:382976694 |
| 23 | GI:311293933 | 57 | GI:382976703 |
| 24 | GI:311293930 | 58 | GI:110609977 |
| 25 | GI:226424281 | 59 | GI:55668309 |
| 26 | GI:342315702 | 60 | GI:55668311 |
| 27 | GI:342315684 | 61 | GI:55668307 |
| 28 | GI:342315690 | 62 | GI:226424287 |
| 29 | GI:342315696 | 63 | GI:40794992 |
| 30 | GI:307827231 | 64 | GI:56485039 |
| 31 | GI:56484996 | 65 | GI:226934561 |
| 32 | GI:377657204 | 66 | GI:342315719 |
| 33 | GI:37223485 | 67 | GI:342315711 |
| 34 | GI:56484965 | 68 | GI:298501351 |
