# Supplementary material for: CpG Distribution and Methylation Pattern in Porcine Parvovirus
Source: PLoS One. 2013 Dec 31;8(12):e85986. doi: 10.1371/journal.pone.0085986 (PMC3877397; doi:10.1371/journal.pone.0085986)
Supplement: Table S2 — Methylation level of the CpG sites in the PPV genome. 2a, Methylation of the CpG sites in PT cells 8 hours post infection. 2b, Methylation of the CpG sites in Cos 7 cells 96 hours post infection. 2c, Methylation of the CpG sites in PPV originated from aborted pig embryos. 2d, Methylation of the CpG sites in the progeny viruses of the in vitro methylated PPV genome. 2e, Methylation of the new CpG sites in the M123 mutant PPV. 2f, Methylation of the progeny viruses after overexpressing of DNMT3a in PT cells. (DOC) [file pone.0085986.s002.doc]

**Table S2.**

2a,

|  | **Positive strand** | | |  | **Negative strand** | | |
| --- | --- | --- | --- | --- | --- | --- | --- |
| **CpG site** | **Position** | **Clones sequenced** | **Methylation** |  | **Position** | **Clones sequenced** | **Methylation** |
| 1 | 28 | 6 | 0 |  | 29 | no data | no data |
| 2 | 38 | 6 | 0 |  | 39 | no data | no data |
| 3 | 46 | 6 | 1 |  | 47 | no data | no data |
| 4 | 48 | 6 | 0 |  | 49 | no data | no data |
| 5 | 50 | 6 | 0 |  | 51 | no data | no data |
| 6 | 55 | 6 | 0 |  | 56 | no data | no data |
| 7 | 57 | 6 | 0 |  | 58 | no data | no data |
| 8 | 59 | 6 | 0 |  | 60 | no data | no data |
| 9 | 68 | 6 | 0 |  | 69 | no data | no data |
| 10 | 79 | 6 | 0 |  | 80 | no data | no data |
| 11 | 147 | 6 | 0 |  | 148 | 4 | 0 |
| 12 | 168 | 6 | 0 |  | 169 | 4 | 0 |
| 13 | 249 | 6 | 0 |  | 250 | 4 | 0 |
| 14 | 299 | 6 | 0 |  | 300 | 4 | 0 |
| 15 | 314 | 6 | 0 |  | 315 | 4 | 0 |
| 16 | 455 | 12 | 1 |  | 456 | no data | no data |
| 17 | 531 | 6 | 0 |  | 532 | no data | no data |
| 18 | 547 | 6 | 0 |  | 548 | no data | no data |
| 19 | 845 | no data | no data |  | 846 | no data | no data |
| 20 | 1017 | no data | no data |  | 1018 | no data | no data |
| 21 | 1079 | 6 | 0 |  | 1080 | no data | no data |
| 22 | 1239 | 6 | 0 |  | 1240 | no data | no data |
| 23 | 1776 | 6 | 0 |  | 1777 | no data | no data |
| 24 | 2051 | 4 | 0 |  | 2052 | 4 | 0 |
| 25 | 2057 | 4 | 0 |  | 2058 | 4 | 0 |
| 26 | 2070 | 4 | 0 |  | 2071 | 4 | 0 |
| 27 | 2120 | 4 | 0 |  | 2121 | 4 | 0 |
| 28 | 2127 | 4 | 0 |  | 2128 | 4 | 0 |
| 29 | 2174 | 4 | 0 |  | 2175 | 4 | 0 |
| 30 | 2191 | 4 | 0 |  | 2192 | 4 | 0 |
| 31 | 2213 | 4 | 0 |  | 2214 | 4 | 0 |
| 32 | 2226 | 4 | 0 |  | 2227 | 4 | 0 |
| 33 | 2291 | 4 | 0 |  | 2292 | 4 | 0 |
| 34 | 2464 | no data | no data |  | 2465 | 2 | 0 |
| 35 | 2467 | no data | no data |  | 2468 | 2 | 0 |
| 36 | 2482 | no data | no data |  | 2483 | 2 | 0 |
| 37 | 2485 | no data | no data |  | 2486 | 2 | 0 |
| 38 | 2494 | no data | no data |  | 2495 | 2 | 0 |
| 39 | 2575 | no data | no data |  | 2576 | 6 | 1 |
| 40 | 2587 | no data | no data |  | 2588 | 6 | 0 |
| 41 | 2624 | no data | no data |  | 2625 | 4 | 0 |
| 42 | 2694 | no data | no data |  | 2695 | 4 | 0 |
| 43 | 2697 | no data | no data |  | 2698 | 4 | 0 |
| 44 | 2893 | no data | no data |  | 2894 | 4 | 0 |
| 45 | 2896 | no data | no data |  | 2897 | 4 | 0 |
| 46 | 2902 | no data | no data |  | 2903 | 4 | 0 |
| 47 | 3004 | no data | no data |  | 3005 | 4 | 0 |
| 48 | 3040 | no data | no data |  | 3041 | 4 | 0 |
| 49 | 3087 | no data | no data |  | 3088 | no data | no data |
| 50 | 3106 | no data | no data |  | 3107 | no data | no data |
| 51 | 3154 | no data | no data |  | 3155 | no data | no data |
| 52 | 3180 | no data | no data |  | 3181 | no data | no data |
| 53 | 3334 | no data | no data |  | 3335 | no data | no data |
| 54 | 4940 | no data | no data |  | 4941 | no data | no data |
| 55 | 4950 | no data | no data |  | 4951 | no data | no data |
| 56 | 4962 | no data | no data |  | 4963 | no data | no data |
| 57 | 4971 | no data | no data |  | 4972 | no data | no data |
| 58 | 4983 | no data | no data |  | 4984 | no data | no data |
| 59 | 4987 | no data | no data |  | 4988 | no data | no data |
| 60 | 4990 | no data | no data |  | 4991 | no data | no data |

2b,

| **Negative strand** | | | |
| --- | --- | --- | --- |
| **CpG site** | **Position** | **Clones sequenced** | **Methylation** |
| 1 | 29 | 2 | 0 |
| 2 | 39 | 2 | 0 |
| 3 | 47 | 2 | 0 |
| 4 | 49 | 2 | 0 |
| 5 | 51 | 2 | 0 |
| 6 | 56 | 2 | 0 |
| 7 | 58 | 2 | 0 |
| 8 | 60 | 2 | 0 |
| 9 | 69 | 2 | 0 |
| 10 | 80 | 2 | 0 |
| 11 | 148 | 6 | 0 |
| 12 | 169 | 6 | 0 |
| 13 | 250 | 4 | 0 |
| 14 | 300 | 4 | 0 |
| 15 | 315 | 4 | 0 |
| 16 | 456 | 4 | 0 |
| 17 | 532 | 4 | 0 |
| 18 | 548 | 4 | 0 |
| 19 | 846 | 3 | 0 |
| 20 | 1018 | 6 | 0 |
| 21 | 1080 | 3 | 0 |
| 22 | 1240 | 3 | 0 |
| 23 | 1777 | 4 | 0 |
| 24 | 2052 | 4 | 0 |
| 25 | 2058 | 4 | 0 |
| 26 | 2071 | 4 | 0 |
| 27 | 2121 | 4 | 0 |
| 28 | 2128 | 4 | 0 |
| 29 | 2175 | 4 | 0 |
| 30 | 2192 | 4 | 0 |
| 31 | 2214 | 4 | 0 |
| 32 | 2227 | 4 | 0 |
| 33 | 2292 | 4 | 0 |
| 34 | 2465 | 4 | 0 |
| 35 | 2468 | 4 | 0 |
| 36 | 2483 | 4 | 1 |
| 37 | 2486 | 4 | 1 |
| 38 | 2495 | 4 | 1 |
| 39 | 2576 | 8 | 0 |
| 40 | 2588 | 8 | 0 |
| 41 | 2625 | 4 | 1 |
| 42 | 2695 | 4 | 1 |
| 43 | 2698 | 4 | 0 |
| 44 | 2894 | 4 | 0 |
| 45 | 2897 | 4 | 0 |
| 46 | 2903 | 4 | 0 |
| 47 | 3005 | 4 | 0 |
| 48 | 3041 | 4 | 0 |
| 49 | 3088 | 3 | 0 |
| 50 | 3107 | 3 | 0 |
| 51 | 3155 | 3 | 0 |
| 52 | 3181 | 3 | 0 |
| 53 | 3335 | 3 | 0 |
| 54 | 4941 | no data | no data |
| 55 | 4951 | no data | no data |
| 56 | 4963 | no data | no data |
| 57 | 4972 | no data | no data |
| 58 | 4984 | no data | no data |
| 59 | 4988 | no data | no data |
| 60 | 4991 | no data | no data |

2c,

|  | **Positive strand** | | |  |  | **Negative strand** | | |
| --- | --- | --- | --- | --- | --- | --- | --- | --- |
| **CpG site** | **Position** | **Clones sequenced** | **Methylation** |  | **CpG site** | **Position** | **Clones sequenced** | **Methylation** |
| 1 | 28 | 5 | 0 |  | 34 | 2465 | 4 | 0 |
| 2 | 38 | 5 | 0 |  | 35 | 2468 | 4 | 0 |
| 3 | 46 | 5 | 0 |  | 36 | 2483 | 4 | 0 |
| 4 | 48 | 5 | 0 |  | 37 | 2486 | 4 | 0 |
| 5 | 50 | 5 | 0 |  | 38 | 2495 | 4 | 0 |
| 6 | 55 | 5 | 0 |  | 39 | 2576 | 4 | 0 |
| 7 | 57 | 5 | 0 |  | 40 | 2588 | 4 | 0 |
| 8 | 59 | 5 | 0 |  |  |  |  |  |
| 9 | 68 | 5 | 0 |  |  |  |  |  |
| 10 | 79 | 5 | 0 |  |  |  |  |  |
| 11 | 147 | 5 | 0 |  |  |  |  |  |
| 12 | 168 | 5 | 0 |  |  |  |  |  |
| 16 | 455 | 2 | 0 |  |  |  |  |  |
| 17 | 531 | 2 | 0 |  |  |  |  |  |
| 18 | 547 | 2 | 0 |  |  |  |  |  |

2d,

| **Negative strand** | | | |
| --- | --- | --- | --- |
| **CpG site** | **Position** | **Clones sequenced** | **Methylation** |
| 1 | 29 | 4 | 0 |
| 2 | 39 | 4 | 0 |
| 3 | 47 | 4 | 0 |
| 4 | 49 | 4 | 0 |
| 5 | 51 | 4 | 0 |
| 6 | 56 | 4 | 0 |
| 7 | 58 | 4 | 0 |
| 8 | 60 | 4 | 0 |
| 9 | 69 | 4 | 0 |
| 10 | 80 | 4 | 0 |
| 11 | 148 | 6 | 0 |
| 12 | 169 | 6 | 0 |
| 13 | 250 | 2 | 0 |
| 14 | 300 | 2 | 0 |
| 15 | 315 | 2 | 0 |
| 16 | 456 | no data | no data |
| 17 | 532 | no data | no data |
| 18 | 548 | no data | no data |
| 19 | 846 | no data | no data |
| 20 | 1018 | no data | no data |
| 21 | 1080 | no data | no data |
| 22 | 1240 | no data | no data |
| 23 | 1777 | 4 | 0 |
| 24 | 2052 | no data | no data |
| 25 | 2058 | no data | no data |
| 26 | 2071 | no data | no data |
| 27 | 2121 | 4 | 0 |
| 28 | 2128 | 4 | 0 |
| 29 | 2175 | 4 | 0 |
| 30 | 2192 | 4 | 0 |
| 31 | 2214 | 4 | 0 |
| 32 | 2227 | 4 | 0 |
| 33 | 2292 | 4 | 0 |
| 34 | 2465 | 4 | 0 |
| 35 | 2468 | 4 | 0 |
| 36 | 2483 | 4 | 0 |
| 37 | 2486 | 4 | 0 |
| 38 | 2495 | 4 | 0 |
| 39 | 2576 | 8 | 0 |
| 40 | 2588 | 8 | 0 |
| 41 | 2625 | 4 | 0 |
| 42 | 2695 | 4 | 0 |
| 43 | 2698 | 4 | 0 |
| 44 | 2894 | 4 | 0 |
| 45 | 2897 | 4 | 0 |
| 46 | 2903 | 4 | 0 |
| 47 | 3005 | 4 | 0 |
| 48 | 3041 | 4 | 0 |
| 49 | 3088 | no data | no data |
| 50 | 3107 | no data | no data |
| 51 | 3155 | no data | no data |
| 52 | 3181 | no data | no data |
| 53 | 3335 | no data | no data |
| 54 | 4941 | 7 | 0 |
| 55 | 4951 | 7 | 0 |
| 56 | 4963 | 7 | 0 |
| 57 | 4972 | 7 | 1 |
| 58 | 4984 | 7 | 0 |
| 59 | 4988 | 7 | 0 |
| 60 | 4991 | 7 | 0 |

2e,

| **Positive strand** | | | |
| --- | --- | --- | --- |
| **New CpG sites** | **Position** | **Clones sequenced** | **Methylation** |
| 1 | 3501 | 5 | 0 |
| 2 | 3516 | 5 | 0 |
| 3 | 3528 | 5 | 0 |
| 4 | 3549 | 5 | 0 |
| 5 | 3555 | 5 | 0 |
| 6 | 3558 | 5 | 0 |
| 7 | 3582 | 5 | 0 |
| 8 | 3585 | 5 | 0 |
| 9 | 3588 | 5 | 0 |
| 10 | 3600 | 5 | 0 |
| 11 | 3690 | 5 | 0 |
| 12 | 3702 | 5 | 0 |
| 13 | 3720 | 5 | 0 |
| 14 | 3732 | 5 | 0 |
| 15 | 3735 | 5 | 0 |
| 16 | 3783 | 5 | 0 |
| 17 | 3792 | 5 | 0 |
| 18 | 3804 | 5 | 0 |
| 19 | 3813 | 5 | 0 |
| 20 | 3840 | 5 | 0 |
| 21 | 3852 | 5 | 0 |
| 22 | 3855 | 5 | 0 |
| 23 | 3876 | 5 | 0 |
| 24 | 3885 | 5 | 0 |
| 25 | 3888 | 5 | 0 |
| 26 | 3903 | 5 | 0 |
| 27 | 3906 | 5 | 0 |
| 28 | 3909 | 5 | 0 |
| 29 | 3918 | 5 | 0 |

2f,

|  | **Positive strand** | | |  | **Negative strand** | | |
| --- | --- | --- | --- | --- | --- | --- | --- |
| **CpG site** | **Position** | **Clones sequenced** | **Methylation** |  | **Position** | **Clones sequenced** | **Methylation** |
| 1 | 28 | 6 | 0 |  | 29 | 6 | 1 |
| 2 | 38 | 6 | 0 |  | 38 | 6 | 0 |
| 3 | 46 | 6 | 0 |  | 47 | 6 | 0 |
| 4 | 48 | 6 | 0 |  | 49 | 6 | 0 |
| 5 | 50 | 6 | 0 |  | 51 | 6 | 0 |
| 6 | 55 | 6 | 0 |  | 56 | 6 | 0 |
| 7 | 57 | 6 | 0 |  | 58 | 6 | 0 |
| 8 | 59 | 6 | 0 |  | 60 | 6 | 0 |
| 9 | 68 | 6 | 0 |  | 69 | 6 | 1 |
| 10 | 79 | 6 | 0 |  | 80 | 6 | 0 |
| 11 | 147 | 6 | 0 |  | 148 | 12 | 1 |
| 12 | 168 | 6 | 0 |  | 169 | 12 | 0 |
| 13 | 249 | 6 | 0 |  | 250 | 6 | 0 |
| 14 | 299 | 6 | 0 |  | 300 | 6 | 0 |
| 15 | 314 | 6 | 0 |  | 315 | 6 | 0 |
| 16 | 455 | 12 | 0 |  | 456 | 6 | 0 |
| 17 | 531 | 6 | 0 |  | 532 | 6 | 0 |
| 18 | 547 | 6 | 0 |  | 548 | 6 | 0 |
| 19 | 845 | 5 | 1 |  | 846 | 6 | 0 |
| 20 | 1017 | 5 | 1 |  | 1018 | 12 | 0 |
| 21 | 1079 | 10 | 0 |  | 1080 | 6 | 0 |
| 22 | 1239 | 5 | 0 |  | 1240 | 6 | 0 |
| 23 | 1776 | 6 | 0 |  | 1777 | 5 | 0 |
| 24 | 2051 | 5 | 2 |  | 2052 | 5 | 0 |
| 25 | 2057 | 5 | 0 |  | 2058 | 5 | 0 |
| 26 | 2070 | 5 | 0 |  | 2071 | 5 | 0 |
| 27 | 2120 | 5 | 0 |  | 2121 | 5 | 0 |
| 28 | 2127 | 5 | 0 |  | 2128 | 5 | 0 |
| 29 | 2174 | 5 | 0 |  | 2175 | 5 | 0 |
| 30 | 2191 | 5 | 0 |  | 2192 | 5 | 0 |
| 31 | 2213 | 5 | 0 |  | 2214 | 5 | 0 |
| 32 | 2226 | 5 | 0 |  | 2227 | 5 | 0 |
| 33 | 2291 | 5 | 0 |  | 2292 | 5 | 0 |
| 34 | 2464 | 6 | 0 |  | 2465 | 5 | 0 |
| 35 | 2467 | 6 | 0 |  | 2468 | 5 | 0 |
| 36 | 2482 | 6 | 0 |  | 2483 | 5 | 0 |
| 37 | 2485 | 6 | 0 |  | 2486 | 5 | 0 |
| 38 | 2494 | 6 | 0 |  | 2495 | 5 | 0 |
| 39 | 2575 | 6 | 0 |  | 2576 | 9 | 0 |
| 40 | 2587 | 6 | 0 |  | 2588 | 9 | 0 |
| 41 | 2624 | 6 | 0 |  | 2625 | 4 | 0 |
| 42 | 2694 | 6 | 1 |  | 2695 | 4 | 0 |
| 43 | 2697 | 6 | 0 |  | 2698 | 4 | 0 |
| 44 | 2893 | no data | no data |  | 2894 | 3 | 0 |
| 45 | 2896 | no data | no data |  | 2897 | 3 | 0 |
| 46 | 2902 | no data | no data |  | 2903 | 3 | 0 |
| 47 | 3004 | no data | no data |  | 3005 | 3 | 0 |
| 48 | 3040 | no data | no data |  | 3041 | 3 | 1 |
| 49 | 3087 | no data | no data |  | 3088 | 5 | 0 |
| 50 | 3106 | no data | no data |  | 3107 | 5 | 0 |
| 51 | 3154 | 4 | 0 |  | 3155 | 5 | 1 |
| 52 | 3180 | 4 | 0 |  | 3181 | 5 | 0 |
| 53 | 3334 | 4 | 0 |  | 3335 | 5 | 0 |
| 54 | 4940 | no data | no data |  | 4941 | no data | no data |
| 55 | 4950 | no data | no data |  | 4951 | no data | no data |
| 56 | 4962 | no data | no data |  | 4963 | no data | no data |
| 57 | 4971 | no data | no data |  | 4972 | no data | no data |
| 58 | 4983 | no data | no data |  | 4984 | no data | no data |
| 59 | 4987 | no data | no data |  | 4988 | no data | no data |
| 60 | 4990 | no data | no data |  | 4991 | no data | no data |
